# Ethyl 1-[(4-acetyl-2-methoxy­phen­oxy)meth­yl]cyclo­propane-1-carboxyl­ate

**DOI:** 10.1107/S1600536809000956

**Published:** 2009-01-14

**Authors:** Ting Tang, Lei Gao, Hong-Sheng Jia, Ya-Ming Wu, Hong-Fei Ma

**Affiliations:** a Department of Applied Chemistry, College of Science, Nanjing University of Technology, Nanjing 210009, People’s Republic of China; b Department of Chemical Engineering, Chien-shlung Institute of Technology, Taicang 215411, People’s Republic of China; c Department of Applied Chemistry, Nanjing College of Chemical Technology, No. 625 Geguan Road, Dachang, Nanjing 210048, People’s Republic of China

## Abstract

In the title compound, C_16_H_20_O_5_, the dihedral angle between the planar rings, *viz*. benzene and cyclo­propane, is 52.1 (2)°. Mol­ecules are connected in the crystal *via* weak inter­molecular C—H⋯O hydrogen bonds, forming chains in the [001] direction.

## Related literature

For details of the synthesis, see: Chen (2008 ▶).
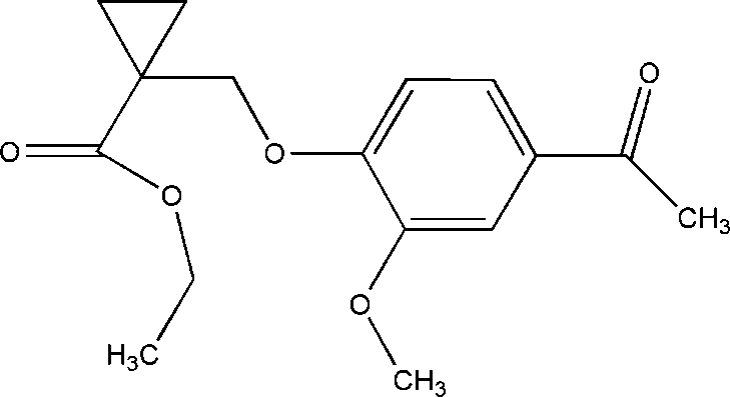


## Experimental

### 

#### Crystal data

C_16_H_20_O_5_
*M* *_r_* = 292.32
Monoclinic, 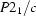

*a* = 12.663 (3) Å
*b* = 8.5020 (17) Å
*c* = 14.676 (3) Å
β = 107.25 (3)°
*V* = 1509.0 (5) Å^3^
*Z* = 4
Mo *K*α radiation
μ = 0.10 mm^−1^
*T* = 298 (2) K
0.20 × 0.10 × 0.10 mm

#### Data collection

Enraf–Nonius CAD-4 diffractometer
Absorption correction: ψ scan (North *et al.*, 1968 ▶) *T*_min_ = 0.981, *T*_max_ = 0.991
2874 measured reflections
2732 independent reflections
1473 reflections with *I* > 2σ(*I*)
*R*_int_ = 0.039
3 standard reflections every 200 reflections intensity decay: 1%

#### Refinement

*R*[*F* ^2^ > 2σ(*F* ^2^)] = 0.062
*wR*(*F* ^2^) = 0.168
*S* = 0.93
2732 reflections
193 parameters
H-atom parameters constrained
Δρ_max_ = 0.22 e Å^−3^
Δρ_min_ = −0.20 e Å^−3^

### 

Data collection: *CAD-4 Software* (Enraf–Nonius, 1985 ▶); cell refinement: *CAD-4 Software*; data reduction: *XCAD4* (Harms & Wocadlo, 1995 ▶); program(s) used to solve structure: *SHELXS97* (Sheldrick, 2008 ▶); program(s) used to refine structure: *SHELXL97* (Sheldrick, 2008 ▶); molecular graphics: *SHELXTL* (Sheldrick, 2008 ▶); software used to prepare material for publication: *SHELXTL*.

## Supplementary Material

Crystal structure: contains datablocks I, global. DOI: 10.1107/S1600536809000956/bh2213sup1.cif

Structure factors: contains datablocks I. DOI: 10.1107/S1600536809000956/bh2213Isup2.hkl

Additional supplementary materials:  crystallographic information; 3D view; checkCIF report

## Figures and Tables

**Table 1:** Hydrogen-bond geometry (Å, °)

*D*—H⋯*A*	*D*—H	H⋯*A*	*D*⋯*A*	*D*—H⋯*A*
C7—H7*A*⋯O1^i^	0.93	2.56	3.329 (4)	140

Symmetry code: (i) 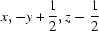.

**Fig. 1.**
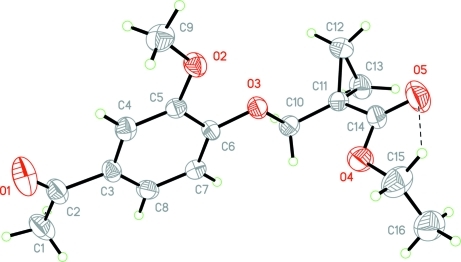
A drawing of the molecular structure of (I), with the atom-numbering scheme. Displacement ellipsoids are drawn at the 40% probability level. Intramolecular C—H···O hydrogen bond is shown as a dashed line.

**Fig. 2.**
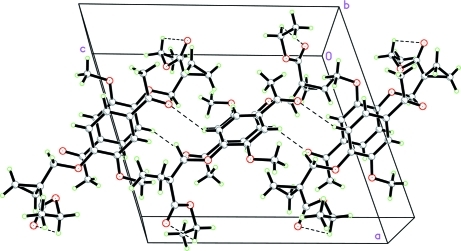
A packing diagram for (I). C—H···O hydrogen bonds are shown as dashed lines.

## supplementary crystallographic information

### Comment

The title compound, (I), is one of the most important intermediates in the synthesis of 7-[(1-aminocyclopropyl)methoxy]-*N*-(1*H*-indol-5-yl)-6-methoxyquinolin-4-amine, which has advantageous pharmacological properties and inhibits the activity of protein tyrosine kinases (Chen, 2008). We report here the crystal structure of (I).

All bond lengths and angles are within expected ranges. Both benzene and cyclopropane rings are planar, and make a dihedral angle of 52.1 (2)° (rings C3···C8 and C11···C13). Molecules are linked together *via* intermolecular C—H···O hydrogen bonds, which may be effective to the stabilization of the crystal structure.

### Experimental

The title compound was synthesized using a method similar to that reported recently (Chen, 2008). The crystals were obtained by evaporating the acetone slowly at room temperature for about 14 d.

### Refinement

H atoms were positioned geometrically, with C—H = 0.93 (aromatic), 0.96 (methyl) or 0.97 Å (methylene), and constrained to ride on their parent atoms, with *U*_iso_(H) = *xU*_eq_(carrier C), where *x* = 1.5 for methyl groups and *x* = 1.2 otherwise. Methyl groups were allowed to rotate about their C—C bonds.

### Crystal data

**Table:**

C_16_H_20_O_5_	*F*(000) = 624
*M**_r_* = 292.32	*D*_x_ = 1.287 Mg m^−^^3^
Monoclinic, *P*2_1_/*c*	Mo *K*α radiation, λ = 0.71073 Å
Hall symbol: -P 2ybc	Cell parameters from 25 reflections
*a* = 12.663 (3) Å	θ = 9–13°
*b* = 8.5020 (17) Å	µ = 0.10 mm^−^^1^
*c* = 14.676 (3) Å	*T* = 298 K
β = 107.25 (3)°	Plate, colorless
*V* = 1509.0 (5) Å^3^	0.20 × 0.10 × 0.10 mm
*Z* = 4	

### Data collection

**Table:**

Enraf–Nonius CAD-4 diffractometer	1473 reflections with *I* > 2σ(*I*)
Radiation source: fine-focus sealed tube	*R*_int_ = 0.039
graphite	θ_max_ = 25.3°, θ_min_ = 1.7°
ω/2θ scans	*h* = 0→14
Absorption correction: ψ scan (North *et al.*, 1968)	*k* = 0→10
*T*_min_ = 0.981, *T*_max_ = 0.991	*l* = −17→17
2874 measured reflections	3 standard reflections every 200 reflections
2732 independent reflections	intensity decay: 1%

### Refinement

**Table:**

Refinement on *F*^2^	Primary atom site location: structure-invariant direct methods
Least-squares matrix: full	Secondary atom site location: difference Fourier map
*R*[*F*^2^ > 2σ(*F*^2^)] = 0.062	Hydrogen site location: inferred from neighbouring sites
*wR*(*F*^2^) = 0.168	H-atom parameters constrained
*S* = 0.93	*w* = 1/[σ^2^(*F*_o_^2^) + (0.06*P*)^2^ + 1.15*P*] where *P* = (*F*_o_^2^ + 2*F*_c_^2^)/3
2732 reflections	(Δ/σ)_max_ < 0.001
193 parameters	Δρ_max_ = 0.22 e Å^−^^3^
0 restraints	Δρ_min_ = −0.20 e Å^−^^3^
0 constraints	

### Fractional atomic coordinates and isotropic or equivalent isotropic displacement parameters (Å^2^)

**Table:**

	*x*	*y*	*z*	*U*_iso_*/*U*_eq_	
O1	0.5800 (2)	0.2003 (4)	0.7367 (2)	0.0809 (10)	
C1	0.7006 (3)	0.3398 (5)	0.6698 (3)	0.0673 (13)	
H1A	0.7445	0.3495	0.7353	0.101*	
H1B	0.6823	0.4426	0.6428	0.101*	
H1C	0.7418	0.2838	0.6348	0.101*	
O2	0.26250 (19)	−0.0087 (3)	0.46428 (15)	0.0472 (6)	
C2	0.5977 (3)	0.2525 (5)	0.6646 (3)	0.0522 (10)	
O3	0.27923 (17)	0.1243 (3)	0.31287 (15)	0.0431 (6)	
C3	0.5143 (3)	0.2248 (4)	0.5700 (2)	0.0403 (8)	
O4	0.1020 (2)	0.3513 (3)	0.20147 (19)	0.0602 (8)	
C4	0.4262 (3)	0.1219 (4)	0.5639 (2)	0.0417 (9)	
H4A	0.4197	0.0740	0.6189	0.050*	
C5	0.3492 (3)	0.0907 (4)	0.4776 (2)	0.0360 (8)	
O5	−0.0032 (2)	0.2019 (4)	0.0853 (2)	0.0883 (11)	
C6	0.3593 (3)	0.1627 (4)	0.3945 (2)	0.0375 (8)	
C7	0.4462 (3)	0.2622 (4)	0.3995 (2)	0.0415 (9)	
H7A	0.4534	0.3086	0.3442	0.050*	
C8	0.5237 (3)	0.2938 (4)	0.4871 (3)	0.0464 (9)	
H8A	0.5823	0.3616	0.4901	0.056*	
C9	0.2450 (3)	−0.0810 (5)	0.5458 (3)	0.0565 (11)	
H9A	0.1861	−0.1562	0.5259	0.085*	
H9B	0.2256	−0.0022	0.5849	0.085*	
H9C	0.3115	−0.1334	0.5817	0.085*	
C10	0.2902 (3)	0.1857 (4)	0.2248 (2)	0.0413 (9)	
H10A	0.3545	0.1404	0.2118	0.050*	
H10B	0.2994	0.2990	0.2292	0.050*	
C11	0.1888 (3)	0.1450 (4)	0.1471 (2)	0.0414 (8)	
C12	0.1746 (3)	−0.0231 (5)	0.1132 (3)	0.0567 (11)	
H12A	0.2312	−0.0979	0.1453	0.068*	
H12B	0.1001	−0.0653	0.0930	0.068*	
C13	0.2038 (3)	0.0959 (5)	0.0513 (2)	0.0590 (11)	
H13A	0.1471	0.1268	−0.0064	0.071*	
H13B	0.2784	0.0942	0.0461	0.071*	
C14	0.0850 (3)	0.2319 (5)	0.1393 (3)	0.0497 (10)	
C15	0.0056 (4)	0.4466 (6)	0.2022 (3)	0.0750 (14)	
H15A	−0.0610	0.3843	0.1777	0.090*	
H15B	0.0113	0.4756	0.2675	0.090*	
C16	−0.0033 (4)	0.5888 (6)	0.1448 (4)	0.0806 (14)	
H16A	−0.0661	0.6490	0.1485	0.121*	
H16B	−0.0123	0.5605	0.0797	0.121*	
H16C	0.0626	0.6506	0.1687	0.121*	

### Atomic displacement parameters (Å^2^)

**Table:**

	*U*^11^	*U*^22^	*U*^33^	*U*^12^	*U*^13^	*U*^23^
O1	0.0617 (18)	0.131 (3)	0.0465 (16)	0.0004 (19)	0.0099 (14)	−0.0115 (18)
C1	0.052 (2)	0.071 (3)	0.068 (3)	−0.009 (2)	0.001 (2)	−0.023 (2)
O2	0.0434 (13)	0.0557 (16)	0.0413 (13)	−0.0115 (13)	0.0106 (10)	0.0021 (12)
C2	0.048 (2)	0.057 (3)	0.045 (2)	0.007 (2)	0.0029 (18)	−0.015 (2)
O3	0.0417 (13)	0.0481 (15)	0.0364 (12)	−0.0089 (12)	0.0065 (10)	0.0034 (12)
C3	0.0379 (19)	0.043 (2)	0.0363 (18)	0.0070 (17)	0.0048 (15)	−0.0084 (17)
O4	0.0503 (15)	0.0526 (17)	0.0738 (18)	0.0038 (14)	0.0125 (13)	−0.0084 (15)
C4	0.0370 (18)	0.046 (2)	0.042 (2)	0.0080 (17)	0.0112 (15)	−0.0006 (17)
C5	0.0375 (18)	0.0311 (18)	0.0394 (18)	0.0050 (16)	0.0113 (15)	−0.0037 (15)
O5	0.0523 (18)	0.089 (2)	0.101 (2)	0.0043 (17)	−0.0115 (17)	−0.026 (2)
C6	0.0354 (18)	0.036 (2)	0.0351 (18)	−0.0015 (16)	0.0019 (14)	−0.0046 (15)
C7	0.045 (2)	0.035 (2)	0.044 (2)	0.0015 (17)	0.0112 (16)	0.0007 (17)
C8	0.041 (2)	0.035 (2)	0.058 (2)	−0.0016 (17)	0.0063 (17)	−0.0061 (18)
C9	0.058 (2)	0.061 (3)	0.057 (2)	−0.006 (2)	0.0264 (19)	0.010 (2)
C10	0.045 (2)	0.041 (2)	0.0385 (19)	−0.0018 (17)	0.0129 (15)	0.0033 (16)
C11	0.050 (2)	0.037 (2)	0.0351 (18)	−0.0027 (17)	0.0087 (15)	0.0003 (16)
C12	0.066 (3)	0.043 (2)	0.056 (2)	−0.002 (2)	0.0093 (19)	−0.0094 (19)
C13	0.071 (3)	0.066 (3)	0.037 (2)	−0.005 (2)	0.0111 (18)	−0.005 (2)
C14	0.042 (2)	0.051 (2)	0.047 (2)	−0.0062 (19)	0.0001 (18)	0.005 (2)
C15	0.061 (3)	0.069 (3)	0.102 (4)	0.020 (2)	0.034 (3)	0.010 (3)
C16	0.060 (3)	0.071 (3)	0.112 (4)	0.007 (3)	0.026 (3)	0.002 (3)

### Geometric parameters (Å, °)

**Table:**

O1—C2	1.227 (4)	C8—H8A	0.9300
C1—C2	1.482 (5)	C9—H9A	0.9600
C1—H1A	0.9600	C9—H9B	0.9600
C1—H1B	0.9600	C9—H9C	0.9600
C1—H1C	0.9600	C10—C11	1.483 (4)
O2—C5	1.352 (4)	C10—H10A	0.9700
O2—C9	1.419 (4)	C10—H10B	0.9700
C2—C3	1.493 (5)	C11—C14	1.482 (5)
O3—C6	1.360 (3)	C11—C12	1.506 (5)
O3—C10	1.438 (4)	C11—C13	1.532 (5)
C3—C8	1.387 (5)	C12—C13	1.478 (5)
C3—C4	1.399 (5)	C12—H12A	0.9700
O4—C14	1.339 (4)	C12—H12B	0.9700
O4—C15	1.468 (4)	C13—H13A	0.9700
C4—C5	1.376 (4)	C13—H13B	0.9700
C4—H4A	0.9300	C15—C16	1.459 (6)
C5—C6	1.405 (5)	C15—H15A	0.9700
O5—C14	1.189 (4)	C15—H15B	0.9700
C6—C7	1.373 (4)	C16—H16A	0.9600
C7—C8	1.393 (5)	C16—H16B	0.9600
C7—H7A	0.9300	C16—H16C	0.9600
			
C2—C1—H1A	109.5	C11—C10—H10A	110.0
C2—C1—H1B	109.5	O3—C10—H10B	110.0
H1A—C1—H1B	109.5	C11—C10—H10B	110.0
C2—C1—H1C	109.5	H10A—C10—H10B	108.4
H1A—C1—H1C	109.5	C14—C11—C10	119.3 (3)
H1B—C1—H1C	109.5	C14—C11—C12	115.5 (3)
C5—O2—C9	118.1 (3)	C10—C11—C12	117.9 (3)
O1—C2—C1	121.3 (3)	C14—C11—C13	114.4 (3)
O1—C2—C3	119.0 (4)	C10—C11—C13	117.0 (3)
C1—C2—C3	119.7 (4)	C12—C11—C13	58.2 (2)
C6—O3—C10	117.3 (2)	C13—C12—C11	61.7 (2)
C8—C3—C4	118.9 (3)	C13—C12—H12A	117.6
C8—C3—C2	121.8 (3)	C11—C12—H12A	117.6
C4—C3—C2	119.3 (3)	C13—C12—H12B	117.6
C14—O4—C15	117.2 (3)	C11—C12—H12B	117.6
C5—C4—C3	120.9 (3)	H12A—C12—H12B	114.7
C5—C4—H4A	119.5	C12—C13—C11	60.0 (2)
C3—C4—H4A	119.5	C12—C13—H13A	117.8
O2—C5—C4	125.3 (3)	C11—C13—H13A	117.8
O2—C5—C6	115.2 (3)	C12—C13—H13B	117.8
C4—C5—C6	119.5 (3)	C11—C13—H13B	117.8
O3—C6—C7	124.9 (3)	H13A—C13—H13B	114.9
O3—C6—C5	115.1 (3)	O5—C14—O4	123.1 (4)
C7—C6—C5	120.0 (3)	O5—C14—C11	125.5 (4)
C6—C7—C8	120.1 (3)	O4—C14—C11	111.4 (3)
C6—C7—H7A	119.9	C16—C15—O4	112.0 (4)
C8—C7—H7A	119.9	C16—C15—H15A	109.2
C3—C8—C7	120.5 (3)	O4—C15—H15A	109.2
C3—C8—H8A	119.7	C16—C15—H15B	109.2
C7—C8—H8A	119.7	O4—C15—H15B	109.2
O2—C9—H9A	109.5	H15A—C15—H15B	107.9
O2—C9—H9B	109.5	C15—C16—H16A	109.5
H9A—C9—H9B	109.5	C15—C16—H16B	109.5
O2—C9—H9C	109.5	H16A—C16—H16B	109.5
H9A—C9—H9C	109.5	C15—C16—H16C	109.5
H9B—C9—H9C	109.5	H16A—C16—H16C	109.5
O3—C10—C11	108.3 (3)	H16B—C16—H16C	109.5
O3—C10—H10A	110.0		
			
O1—C2—C3—C8	173.4 (4)	C2—C3—C8—C7	178.2 (3)
C1—C2—C3—C8	−7.6 (5)	C6—C7—C8—C3	0.3 (5)
O1—C2—C3—C4	−9.0 (5)	C6—O3—C10—C11	−173.7 (3)
C1—C2—C3—C4	170.0 (3)	O3—C10—C11—C14	74.4 (4)
C8—C3—C4—C5	−0.8 (5)	O3—C10—C11—C12	−74.2 (4)
C2—C3—C4—C5	−178.5 (3)	O3—C10—C11—C13	−140.7 (3)
C9—O2—C5—C4	3.6 (5)	C14—C11—C12—C13	104.1 (4)
C9—O2—C5—C6	−177.7 (3)	C10—C11—C12—C13	−106.1 (4)
C3—C4—C5—O2	178.9 (3)	C14—C11—C13—C12	−106.0 (4)
C3—C4—C5—C6	0.2 (5)	C10—C11—C13—C12	107.5 (4)
C10—O3—C6—C7	3.6 (5)	C15—O4—C14—O5	0.7 (6)
C10—O3—C6—C5	−175.5 (3)	C15—O4—C14—C11	−179.3 (3)
O2—C5—C6—O3	1.2 (4)	C10—C11—C14—O5	−173.6 (4)
C4—C5—C6—O3	179.9 (3)	C12—C11—C14—O5	−24.2 (6)
O2—C5—C6—C7	−178.1 (3)	C13—C11—C14—O5	40.7 (5)
C4—C5—C6—C7	0.7 (5)	C10—C11—C14—O4	6.4 (5)
O3—C6—C7—C8	179.9 (3)	C12—C11—C14—O4	155.8 (3)
C5—C6—C7—C8	−1.0 (5)	C13—C11—C14—O4	−139.3 (3)
C4—C3—C8—C7	0.6 (5)	C14—O4—C15—C16	−96.6 (4)

### Hydrogen-bond geometry (Å, °)

**Table:**

*D*—H···*A*	*D*—H	H···*A*	*D*···*A*	*D*—H···*A*
C7—H7A···O1^i^	0.93	2.56	3.329 (4)	140
C15—H15A···O5	0.97	2.32	2.678 (6)	101

Symmetry codes: (i) *x*, −*y*+1/2, *z*−1/2.
